# Tumor-Specific Chromosome Mis-Segregation Controls Cancer Plasticity by Maintaining Tumor Heterogeneity

**DOI:** 10.1371/journal.pone.0080898

**Published:** 2013-11-25

**Authors:** Yuanjie Hu, Ning Ru, Huasheng Xiao, Abhishek Chaturbedi, Neil T. Hoa, Xiao-Jun Tian, Hang Zhang, Chao Ke, Fengrong Yan, Jodi Nelson, Zhenzhi Li, Robert Gramer, Liping Yu, Eric Siegel, Xiaona Zhang, Zhenyu Jia, Martin R. Jadus, Charles L. Limoli, Mark E. Linskey, Jianhua Xing, Yi-Hong Zhou

**Affiliations:** 1 Department of Biological Chemistry, University of California Irvine, Irvine, California, United States of America; 2 Department of Neurological Surgery, University of California Irvine, Irvine, California, United States of America; 3 National Engineering Center for Biochip at Shanghai, Shanghai, China; 4 Diagnostic & Molecular Health Care Group, Veterans Affairs Medical Center, Long Beach, California, United States of America; 5 Department of Biological Sciences, Virginia Polytechnic Institute and State University, Blacksburg, California, United States of America; 6 State Key Laboratory of Oncology in South China and Collaborative Innovation Center for Cancer Medicine, Sun Yat-sen University Cancer Center, Guangzhou, China; 7 Ziren Research LLC, Irvine, California, United States of America; 8 Department of Biostatistics, University of Arkansas for Medical Sciences, Little Rock, Arkansas, United States of America; 9 Guizhou Provincial Key Laboratory of Computational Nano-Material Science, Guizhou Normal College, Guiyang, China; 10 Department of Statistics, University of Akron, Akron, Ohio, United States of America; 11 Department of Family and Community Medicine, Northeast Ohio Medical University, Rootstown, Ohio, United States of America; 12 Department of Pathology & Laboratory Medicine, University of California Irvine, Irvine, California, United States of America; 13 Department of Radiation Oncology, University of California Irvine, Irvine, California, United States of America; Cleveland Clinic, United States of America

## Abstract

Aneuploidy with chromosome instability is a cancer hallmark. We studied chromosome 7 (Chr7) copy number variation (CNV) in gliomas and in primary cultures derived from them. We found tumor heterogeneity with cells having Chr7-CNV commonly occurs in gliomas, with a higher percentage of cells in high-grade gliomas carrying more than 2 copies of Chr7, as compared to low-grade gliomas. Interestingly, all Chr7-aneuploid cell types in the parental culture of established glioma cell lines reappeared in single-cell-derived subcultures. We then characterized the biology of three syngeneic glioma cultures dominated by different Chr7-aneuploid cell types. We found phenotypic divergence for cells following Chr7 mis-segregation, which benefited overall tumor growth *in vitro* and *in vivo*. Mathematical modeling suggested the involvement of chromosome instability and interactions among cell subpopulations in restoring the optimal equilibrium of tumor cell types. Both our experimental data and mathematical modeling demonstrated that the complexity of tumor heterogeneity could be enhanced by the existence of chromosomes with structural abnormality, in addition to their mis-segregations. Overall, our findings show, for the first time, the involvement of chromosome instability in maintaining tumor heterogeneity, which underlies the enhanced growth, persistence and treatment resistance of cancers.

## Introduction

According to Nowell's initial clonal evolution hypothesis [1], cancer development is an evolutionary and ecological process, in many ways resembling Darwinian evolution [2]. This hypothesis is supported by prediction of tumor progression with genetic clonal diversity in esophageal adenocarcinoma [3], and now has been widely accepted as an explanation for the tumor heterogeneity observed in most cancers at the time of clinical diagnosis, at both the original and metastatic sites [4], [5]. The concept of cancer as an evolutionary process, with tumors having genetically and phenotypically diverse cell subpopulations is consistent with the recent cancer stem cell model, which emphasizes the importance of cancer having a cell type capable of generating other cell types in a unidirectional manner [6]–[9]. However, the finding of phenotypic inter-conversion among three subpopulations of cells within breast cancer cell lines, leading to a cell population equilibrium [10] revealed the ability of cancer to recover biological diversity from more than just the stem-like cell subpopulation. Such ability to recover equilibrium conditions after a disturbance is a feature characteristic of an established, well-balanced ecosystem. The question remains whether, and how, cancer cell phenotypic transition manifests as an inherited feature.

Accumulating evidence supports the notion that mitotic errors cause chromosome instability, which drives cancer evolution, with natural selection acting at the cancer ecology level to avoid cytogenetic chaos. Apparently, the non-random distribution of chromosomal gains and losses seen in specific tumor types is a combined effect of chromosome instability and selection for specific phenotypes from among massive changes of the transcriptome [11]–[15]. Gliomas are primary malignant brain tumors having astrocytic and/or oligodendroglial features of varying malignancy. The highest grade, unfortunately the most commonly seen glioma, is glioblastoma multiforme (GBM, grade IV), morphologically, genetically, and cytogenetically heterogeneous, and uniformly fatal due its rapid cellular proliferation and strongly invasive behavior [16]–[19]. It is known that alteration of chromosome 7 (Chr7) copy number occurs in both high- and low-grade gliomas and that these changes appear to be associated with invasive and proliferative cell phenotypes [20]–[24]. Here we report studies of Chr7-aneuploidy-related cell diversity and the role of Chr7 mis-segregation (Chr7-MS) in maintaining the phenotypic diversity of glioma cell subpopulations, which generates a synergistic effect on overall tumor growth.

## Materials and Methods

### Ethics Statement

Frozen and fresh glioma specimens were provided by the Tissue Banks of University of California, Irvine and University of Arkansas for Medical Sciences, with Institutional Review Board approval.

### Animal work and subcutaneous (s.c.) and intracranial (s.c.) xenografts

The animal work was approved by Animal Care and Use Committee (IACUC) of University of California, Irvine. For studies using intracranial (i.c.) xenografts, glioma cells (1×10^5^/3 µl DMEM/F12) were injected into the frontal lobe of 4–6 week old, female, nude mice (stain NCrNu-M, Taconic, Hudson, NY), following IACUC approved surgical procedures. After i.c. implantation, mice were observed daily and periodically weighed for moribund signs (hunchback posture, marked weight loss and gait impairment). Mice were euthanized when they developed brain-damage symptoms (ataxia, hemiparesia, etc) and/or 20% body weight loss, and the following day was record as the survival date for survival analysis.

For studies using subcutaneous (s.c.) xenografts, cells (1×10^6^ cells/50 µl DMEM/F12) were subcutaneously injected into nude mice, anterior to their right and left thighs, on both sides. Tumor measurements were taken every 3–4 days after implantation, and tumor volume was calculated using the formula V = (L*W^2^)/2 (L, length; W, width). Mice were euthanized at a predetermined time of the experiment or when tumor volume exceeded 1.5 cm^3^.

### Glioma primary cultures and cell lines

Fresh human glioma tissues were dissociated enzymatically (0.05% trypsin-EDTA for 30–45 min at 37°C), disrupted mechanically (passing through a glass pipette in DMEM/F12 containing 0.10 mg/ml DNase and 10% serum), and cultured in both collagen-coated (3–4 µg/cm^2^) culture dishes in DMEM/F12 supplemented with 5% fetal bovine serum, designated as serum adherent (SA) culture conditions, and agar (1%)-coated culture dishes in DMEM/F12 supplemented with epidermal growth factor (EGF, 20 ng/ml), basic fibroblast growth factor (FGF, 10 ng/ml), and 1–5% B27 (Invitrogen, Carlsbad, CA), designated as neural sphere (NS) culture conditions. The multicellular glioma spheres formed in NS culture conditions were passed into fibronectin (1 µg/cm^2^) coated dishes in the same culture medium before freezing or subjecting to FISH analysis.

The human glioma cell lines (A172, LN229, LG11, T98G, U251, and U87) were obtained from the Department of Neuro-Oncology, the University of Texas M.D. Anderson Cancer Center. The genetic profiles (7-STR markers provided by IDEXX RADIL, Columbia, MO) used by this study were identical or highly similar to the genetic profiles reported for each cell line (Table S1). A172 reported here was originally named as D54, but carries genetic profiles suggesting a variant of A-172 reported by American Type Culture Collection (ATCC). U251 reported here was originally named as U251HF, with genetic profile suggesting a variant of U251, compared to U251 in NCI-60 Cancer Cell Line Panel. The comparisons of U251 variants (Table S2) were provided by Beth Bauer (IDEXX RADIL).

All glioma cell lines (parental) were cultured in SA conditions. The derived SA and NS clones were established from single colonies formed in 0.3% soft agar on top of a layer of bottom agar (0.5%) in DMDM/F12 supplemented with 5% bovine serum or EGF/bFGF/B27 as for NS cultures, picked by a glass pipette, and expanded in SA or NS conditions. For U251 the same homozygous mutations of PTEN [E242fs*15 (723 724 insTT)] and TP53 (R273H) in parental and SA and NS-subcultures were identified by Mariam Youssef, Nirvi Shah and Anthony Wong (UC Irvine).

### Fluorescence in situ hybridization (FISH)

Metaphase-spread slides were obtained by exposing 80% confluently growing cells to nacadozole solution (100 µg/ml final, Sigma) for 1 hour. Then the cells were trypsinized (0.25% trypsin/EDTA, Invitrogen) to collect cell pellets, which were treated with a hypotonic solution (phosphate buffer) for 5 minutes at 37°C. The cell pellets were fixed (methanol:glacial acetic acid = 3∶1) for at least 30 minutes. Finally, the cell suspensions were dropped onto slides to get metaphase chromosome spreads. The standard B-banding was done for the slides as they were done as (same time) treatment with trypsin, and stained with Giemsa stain (Invitrogen). FISH was performed on metaphase spreads and frozen tumor sections (7 µm) using Direct Labeled Fluorescent DNA Probe Kits with CEP X/CEPY, EGFR/CEP 7 and PTEN/CEP10 (Abbott Molecular Inc. Des Plaines, IL). Hybridization, washing, and counterstaining were performed according to the manufacturer's instructions. 250–300 cells per sample were counted under a fluorescent microscope with a 100×lens.

### Lentivirus infection

Infectious lentivirus was produced by co-transfection of the lentiviral vector plasmid pGIPZ-Empty and pTRIPZ-Empty (Open Biosystems) with packaging plasmid psPAX2 and envelope plasmid pCMV-VSVG in HEK-293T cells, following the manufacturer's protocol.

### Immunofluorescence analyses of i.c. xenografts

The cryosections (7–8 µm) of mouse brains with i.c. xenografts were mounted for direct observation of fluorescence expressed by the RFP and GFP-labeled tumor cells using 2× and 20× lenses of a Keyence BZ8100 fluorescence microscope, after nuclear staining with DAPI. Adjacent cryosections were subjected to immunofluorescence analyses using 15 µg/ml rabbit BMI1 (ab38432, Abcam), 15 µg/ml mouse CD133 (130-090-422, Miltenyi Biotec), 10 µg/ml mouse CD31 (CBL1337, Chemicon), 15 µg/ml goat GFAP (sc-6170, Sana Cruz), 10 µg/ml rabbit MELK (A01390, GenScript), and 15 µg/ml mouse SPARC (sc-73051, Sana Cruz) primary antibodies, followed by appropriate secondary antibody, donkey anti-mouse, rabbit, rat, or goat Alexa Fluor 350 (blue), Alexa Fluor 488 (green), and Texas Red (Invitrogen), following the immunofluorescence process as described previously [25]. The tissue sections were mounted with ProLong Gold antifade reagent (Invitrogen), viewed with a 40×lens of a fluorescence microscope and imaged with a spot camera. Co-localization images were acquired and analyzed using a Nikon two-laser (HeNe and Argon) PCM 2000 Confocal System on an Eclipse E800 Microscope with 100× objective (Melville, NY)_ENREF_20.

### Neural stem cell differentiation assay

Glioma cells (5–10×10^3^) maintained in neural sphere culture conditions were seeded into 8-well slides pre-coated with fibronectin (10 ng/ml) for overnight culture in original medium (undifferentiation), or in poly-L-lysine (15 µg/ml) coated wells in DMEM/F12 containing 1% FBS for 7–10 days of culture (differentiation); a half volume of fresh medium was added every 3 days, prior to fixation for immunofluorescence analyses. Cells were fixed with 4% PFA and blocked with 10% donkey serum. Primary antibodies (rabbit Nestin (1∶1000) from Millipore (AB5922), mouse MAP2 (1∶200) from Abcam (ab11267), and goat GFAP (1∶300) from Sana Cruz (sc-6170), mouse Beta Tubulin III (1∶200) from Chemcon (MAB1637)), mouse CD133 (1∶50) from Miltenyi Biotec (130-090-422), rabbit MELK (1∶200) from GenScript USA (A01390), and rabbit BMI (1∶200) from Abcam were incubated with cells overnight at 4°C and developed using AlexaFluor secondary antibodies (mouse or rabbit Alexa Fluor 488 nm and 594 nm (1∶200) from Invitrogen).

### Real-time comparative quantitative polymerase chain reaction (CQ-PCR) and quantitative reverse transcription (qRT-) PCR

DNA samples from frozen glioma specimens were isolated using a DNeasy kit (QIAGEN, Valencia, CA). CQ-PCR standard (product CQ101) and PCR primers to quantify *EGFR* and three reference genes in 2q34 (*SPAG16*), 3p14.3 (*ERC2*), and 5q31.2 (*SPOCK1*) were from Ziren Research LLC (Irvine, CA). It is a recombinant DNA containing PCR fragments of *EGFR* and reference genes in one piece to determine CNV as described previously [26]. Real-time PCR was carried out using FAST-START SYBR-Green I Master Mix (Roche).

Total RNA (∼1 µg) extracted using Ultraspec (Biotecx) from SA and NS-adherent cultures, after a 24-hour culture in basal medium, was converted into cDNA using 5 units of Superscript II reverse transcriptase (Invitrogen). The cDNA samples were diluted and quantified for gene expressions by real-time qRT-PCR (SYBR Green I) using a single standard for marker and reference genes [27], normalized to *ACTB*. Quantification of *GAPDH* was also performed to compare with gene of interest. The primer sequences for genes in qRT-PCR and CQ-PCR are available from Ziren Research LLC (www.zirenresearch.com) upon request.

### Comparative genome hybridization (CGH)

DNA (1.5 µg) samples of glioma cells and control (a pool of six normal human blood DNA samples) were differentially labeled with Cy5 and Cy3-dUTP, respectively, purified and then hybridized to an Agilent Human Genome CGH 244 k Microarray. The data were statistically analyzed and visualized using two independent methods, including Agilent Genomic Workbench 6.5 (Agilent) with Z-score algorithm and a program written in R (http://www.r-project.org/), which detected the same chromosomal aberrations. The threshold of the Z-score used for the Agilent method was set to 4.

### Gelatin zymography, enzyme immunometric assays, Western blotting, and immunocytofluorescence

Proteins in 24-hour conditioned cell culture media were precipitated with 4 volumes of cold acetone, spun immediately at 14,000 rpm for 5 minutes at 4°C, and resuspended in radioimmunoprecipitation assay buffer (RIPA) containing Protease Inhibitor Cocktail (Roche). The same amount of conditioned medium protein was used to run gelatin zymography. Conditioned medium was subjected to enzyme-linked immunosorbent assay (ELISA) for VEGFA (VEGF-165) and SPP1 (Osteopontin) using kits from Assay Designs (Ann Arbor, MI), and PTN from R&D Systems (Minneapolis, MN). Sonicated whole-cell lysate in RIPA was used to perform Western blotting, with antibodies of EGFR from Cell Signaling, and Actin from EMD Bioscience. Cells seeded on Poly-L-lysine or Fibronectin coated 8-well chamber slides, 2×10^4^ cells per chamber, and incubated overnight, were fixed with 4% paraformaldehyde in PBS, with a brief permeabilization in 0.1% triton x-100, and an overnight incubation with primary EGFR antibody at 4°C. The immunocytofluorescence signal was detected after incubation with Alexa Fluor® 594 secondary antibody.

### Soft agar colony formation assay

800–1000 cells were mixed with 1 ml of 0.3% soft agar in DMEM/F12 supplemented with 5% bovine serum or a mitogen supplement for NS cultures as detailed above, spread onto hardened 0.5% soft agar in the same medium (1 ml per well in four corner wells of a 6-well plate). 1 ml of the same medium was added 2 and 3 weeks later and colony numbers were counted 4 weeks later under a microscope with 4×lens.

### Statistical analysis

MANOVA analysis was used in conjunction with ternary plots (http://www.davidgraham.org.uk) to compare GBM to OG samples for percentages of cells bearing one copy, two copies, or ≥3 copies of Chr7. Stem-like cell- and nonstem-like cell-enriched subcultures were compared for differences in gene expression, ELISA, and zymography data by means of 2-sample equal-variance t-tests. Overall survival of mice bearing intracranial glioma xenografts was estimated via Kaplan-Meier survival curves, then compared for differences using a stratified Cox regression model in order to adjust for potential variation (“Day effects”) between different experiments. SAS versions 9.2 and 9.3 (The SAS Institute, Cary, NC) were used for all analyses and *P*<0.01 was used as the significance value to adjust for multiple comparisons without overinflating Type II error.

### Mathematical modeling

#### Mathematical model Construction

Denote x1, x2, x3, x4, x5 as the abundances of cells with 1–5 copies of Chr7. We neglected cells with 6 copies, assuming that they were anaphase stages of 3-copy Chr7 cells. We didn't expect that the results below would be significantly affected by this assumption since the percentage of the 6-copy cells is low in all the measurements. For simplicity we also assume that the mis-segregation rates of normal and abnormal Chr7 are the same. For STICs (2Chr7:1n,1d), we assume the cells can either divide symmetrically, or have one Chr7 mis-segregation, as summarized below 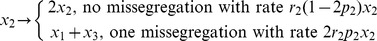



Similarly for TMCs (3Chr7:2n,1d), we have
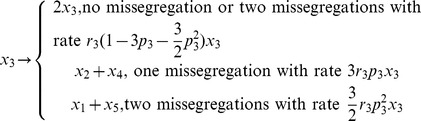



The parameters *r*_i_ is the growth rate constant of species *i*, p_2_ and p_3_ refer to the probabilities of asymmetric (mis-) segregation of one pair of Chr7 in the STICs and TMCs per cell division, respectively. In general these probabilities depend on **x**, but for simplicity we neglect such possible dependence. Notice that for the TMCs, double mis-segregation of two pairs of Chr7 can result in either 1+5 or 3+3, for which we assume an equal probability. For cells with other Chr7 copy numbers, since their percentages are very low, we neglected the even smaller contributions of possible chromosome mis-segregation events. The governing rate equations are
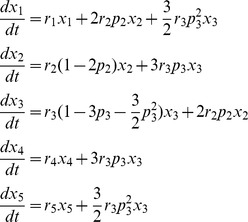



For convenience of discussion we also denote the percentage of each subpopulation α at a given time point i as 
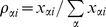
. These quantities were what measured experimentally using FISH.

We consider three cases

- No mis-segregation, i.e., p_2_ = p_3_ = 0 SCs and MCs have no direct mutual influence
- Mis-segregation exists, STICs and TMCs have no direct mutual influences
- Missegragation exist, STICs partially inhibit the growth of TMCs, which is modeled by a Hill function as 
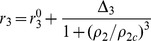
, and MCs activate the growth of SCs, which is modeled as 
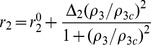
, where 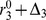 and 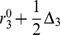 are the growth rate constant of TMC when STIC percentage 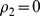 and 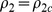, respectively, and 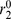 and 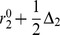 are the growth rate constant of STIC when TMC percentage 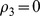
and 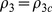, respectively. We actually also consider the case with Hill coefficient 4 instead of 2, but the result shows no significant change

Numerical method

The above ordinary differential equations were solved using Matlab. At the beginning of each passage, we rescale 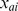 so 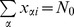. Experimentally 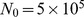. For our mathematical modeling the exact number of N_0_ does not affect the results. For passage 1, we used the values of ρ used experimentally as the initial values. For subsequent passages, we used the values ρ calculated at the end of previous passage as the initial values.

For each case, the best set of parameters was obtained by minimizing 
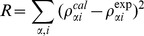
where 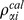 and 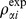 refer to the calculated and measured percentages of subpopulation α at the end of passage i, respectively. We used the down-hill simplex approach [28] to perform the minimization. With each best set of parameters, we predicted the doubling times.

## Results

### Tumor heterogeneity specified by Chr7-CNV commonly exists in high and low grade gliomas

**Figure 1:**
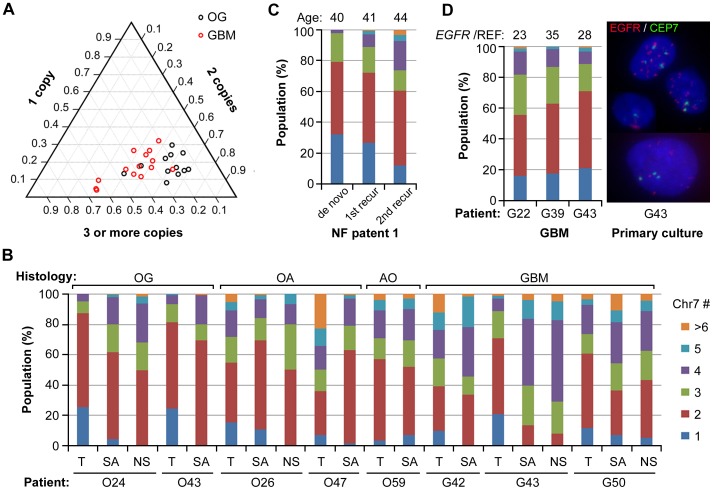
Proportion of cells with Chr7 number variation in high- and low-grade gliomas and glioma primary cultures. **A**, ternary plot of population proportions with 1 copy (deletion), 2 copies (normal), and 3 or more (amplification) copies of Chr7, based on CEP7 signals in Ffuorescent *in situ* hybridization (FISH) of 14 glioblastoma multiformes (GBMs, *Red triangle*) and 12 oligodendroglial tumor (OGs, *black square*), *P* = 0.0012 from MANOVA analysis. **B**, comparison of tumor heterogeneity with regard to chromosome 7 (Chr7) aneuploidy in the original tumor (T) and corresponding 3–4 week-old primary cultures under serum adherent (SA) or neural sphere (NS) culture conditions. **C–D**, patterns of cells with Chr7-CNV in sequential and high-EGFR amplified GBMs. Representative FISH pictures of cells carrying 1–4 copies of Chr7 with focal EGFR amplification.

To determine Chr7 copy number variation (CNV) at the cell subpopulation level, we performed fluorescent *in situ* hybridization (FISH), with dual probes for the *EGFR* gene and the centromeric region of chromosome 7 (CEP7). We examined GBM and oligodendroglial tumor (OT), the second-most-common group of gliomas, characterized by oligodendroglial features. OT includes oligodendroglioma (OG, grade II), oligoastrocytoma (OA, grade II); and anaplastic oligodendroglioma (AO, grade III), based on criteria of the World Health Organization. The number of Chr7 centromeres per nucleus, detected by the FISH CEP7 probe, was determined by counting over 250 cells per tumor, and these data were used to establish the level of tumor heterogeneity with regard to Chr7-CNV. We then performed a comparison of differences in the equilibrium state for tumor heterogeneity based on Chr7-CNV data from 14 GBMs and 12 OGs. There was a significantly higher percentage of cells carrying more than 2 copies of Chr7 (amplification) in GBM compared to OG (*P*<0.0005) (Figure 1A). In contrast to OG, the Chr7-CNV in OA and AO was close to that of GBM, with representative data shown in Figure 1B.

To determine if Chr7 CNV-characterized tumor cell populations are viable and contributing to the clonal diversity within each tumor, we examined short-term (4–6 weeks with 1–2 passages) primary cultures under serum adherent (SA) and/or neural sphere (NS) culture conditions. We found subpopulation cells with Chr7-CNV in all examined glioma primary cultures (Figure 1B). There was a higher percentage of cells with Chr7-amplification in cultures from GBM than in those from OG. In both cases, the percentage of cells with Chr7-ampliciation was higher in primary cultures than in the corresponding tumor. For AO, which is a more progressive type of OT, we also observed a similar Chr7-amplification in the tumor and in its derived primary culture. Interestingly, in oligo-astro mixed OT, named “OA”, the equilibrium in cell composition for Chr7-heterogeneity in the original tumors was similar to that in GBM. However, in OA-derived primary cultures, we observed heterogeneity strikingly resembling that of OG, with a majority of cells having two Chr7 copies and fewer than 40% of cells carrying three or more copies of Chr7 (Figure 1B).

We then compared the patterns of Chr7-heterogeneity in OG or GBM with recurrent and *de novo* status, and found no correlation. However, there were incremental increases in the percentage of cells with Chr7-amplification in sequential GBMs (i.e. tumors sampled over time at recurrence or progression) from a patient with neurofibromatosis (Figure 1C). This is consistent with the analysis above showing a faster growth capability for cells with Chr7-amplificaiton.

A direct molecular consequence of Chr7 amplification is the amplification of the oncogene EGFR residing within it, which confers a growth advantage. Focal amplification of EGFR is commonly seen in the classical subtype of GBM [29]. We thus compared Chr7-CNV with EGFR-CNV, determined by real-time comparative quantitative PCR. We found a balanced *EGFR* relative to reference genes (ratio 0.7–1.3, given a 20–30% variation in quantification) in all oligodendroglial tumors (n = 17) and in 53% of the GBMs (n = 51), and a low level of *EGFR* amplification (ratio 1.4–2) in 23.5% of GBMs, all well correlated with calculated *EGFR* levels, based on the percentage of cells and their Chr7 number. In the remaining 23.5% of GBM, we found a high level of *EGFR* amplification (ratio between 5–48), which was verified by EGFR/CEP7 FISH to be focal EGFR amplification (Figure 1D). In EGFR (focal) amplified GBMs, the pattern of Chr7-tumor heterogeneity was found to be similar to that in GBMs without EGFR (focal) amplification.

Taken together, we observed a substantial level of tumor heterogeneity, with cells showing Chr7-CNV commonly occurring in both low- and high-grade gliomas. Monosomy of chromosome 10 is also a chromosome instability functionally related to tumor malignancy [30] and occurs in about 80% of GBM tumors. When present, monosomy 10 is shown homogenously, in contrast to the heterogeneity seen for Chr7. This difference suggests that there must be an active process for maintaining Chr7 heterogeneity in tumors, which we have identified to be Chr7-MS. To further study this process we examined Chr7-MS and Chr7-CNV in established glioma cell lines.

### Chr7-MS is involved in maintaining cell heterogeneity in established glioma cell lines

**Figure 2:**
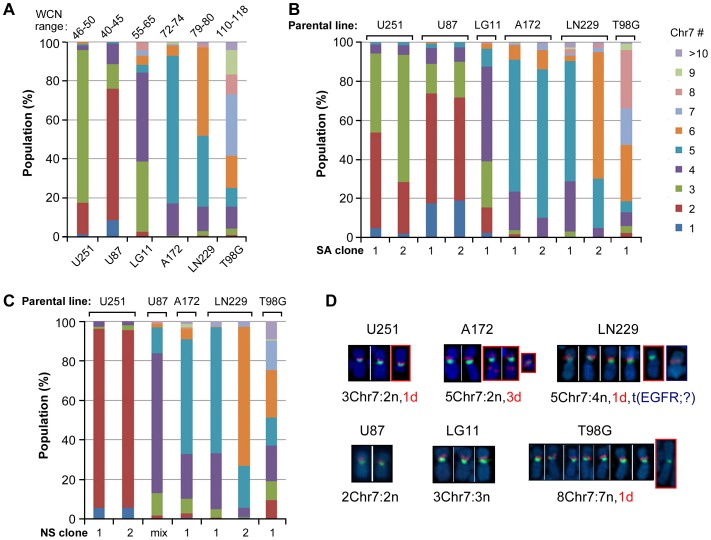
Equilibrium of heterogeneity in cells with Chr7-CNV in established glioma cell lines and their clonal subcultures. **A–B**, percentage of cells with Chr7-CNV in glioma cell lines, and their SA and NS subcultures from single (e.g. 1, 2) or mixed (mix) soft-agar colonies, respectively. Whole chromosome numbers (WCN) ranging near the mode were found in >50% of cells in each glioma cell line. **D**, FISH pictures showing normal (n) and derivative (d) Chr7 and the unknown (?) chromosome carrying a translocated EGFR from a representative metaphase cell for each cell line. The chromosome is shown by DAPI (blue), centromere and EGFR are shown by FISH probes for CEP7 (green) and EGFR (red).

We performed B-banding using Giemsa stain on chromosome spreads of six human malignant glioma cell lines and determined the range of whole chromosome numbers (WCN) clustered around the mode based on more than 7 cells. EGFR/CEP7 FISH were performed and counts of CEP7 signals in more than 250 interphase cells were used to determine the percentage of cells carrying different numbers of Chr7 (Figure 2A). All glioma cell lines showed co-existence of diverse cell subpopulations based on Chr7-CNV, with a significant percentage of cells carrying 2-, 3-, and 4-copies of Chr7 with a near-diploid karyotype (U251, U87 and LG11), 4-, 5-, and 6-copies of Chr7 in near-triploids/tetraploid (A172 and LN229), and 6-, 7-, and 8-copies of Chr7 in near-pentaploid karyotypes (T98G).

Under SA-culture conditions that have been used to culture these glioma cell lines, we established subcultures from single-cell plating or single soft-agar colonies of the glioma cell lines, which we named SA clones (SA1, SA2, etc). FISH showed re-appearance of Chr7-cell heterogeneity in each of the SA clones (Figure 2B), retaining characteristic features of Chr7 and *EGFR* amplification and/or translocation that were found in their parental cultures (Figure 2D). Interestingly, the percentage of those cells that had been in the majority in the parental culture decreased in the clonal SA subcultures. The exception was LN229, which was originally composed of two subpopulations of cells nearly equal in percentage. Evidently, tumor heterogeneity was maintained in the established glioma cell line by Chr7-MS. It also indicates that an established cell line is a cultured ecosystem with cell subpopulations reaching certain equilibrium over time. We then wondered if by changing culture conditions we could change the heterogeneity equilibrium, such as to allow a previously minority cell subpopulation to become dominant under new culture conditions. If that proved to be the case, we would be able to vary culture conditions to obtain sufficient numbers of the various minority cells for further study of their phenotypes and contributions to overall growth.

We exposed glioma cell lines to NS culture conditions, which were originally developed for culturing neural stem cells and then modified for enriching glioma cells expressing neural stem-like cell features [31], [32]. We found that after a month's culture in NS conditions, during which there was a massive dying of cells, (with the dead cells repeatedly removed by passing cells back and forth between non-adherent and fibronectin-mediated adherent conditions in NS medium), a minority cell subpopulation in the parental line came to dominate the NS subcultures. We further established clonal NS subcultures from single colonies formed in soft-agar prepared using NS medium, and named these “NS clones” (NS1, NS2, etc). Figure 2C shows representative FISH data of NS subcultures of glioma cell lines. These data show re-appearances of Chr7-cell heterogeneity from clonal NS subcultures.

It took 4 weeks for colonies to form in soft agar, prior to their transfer to SA or NS culture conditions for further growth. After transfer, it took about 2 weeks to obtain enough cells (2×10^5^) for FISH analysis. The time required to re-establish culture heterogeneity from a single cell was less than 18 cell divisions, which took about 6–7 weeks. Re-gaining of heterogeneous cell cultures with Chr7-CNV in clonal SA and NS subcultures from all studied glioma cell lines demonstrated that Chr7-MS could happen in any subpopulation cell to drive tumor population diversity, while the culture conditions determined the heterogeneity equilibrium of the tumor cell types.

Dramatic changes in the equilibrium of cell subpopulations between SA and NS cultures were noted for the two glioma cell lines with near diploid karyotypes, U251 and U87. Whether chromosome instability, here Chr7-MS, is an important mechanism for generating/maintaining tumor/culture cell heterogeneity, to the benefit of overall tumor growth, was the question we attempted to address next by focusing on the characterization of cell subpopulations of U251, as described below.

### Distinct karyotypes in three cell subpopulations of U251

**Figure 3:**
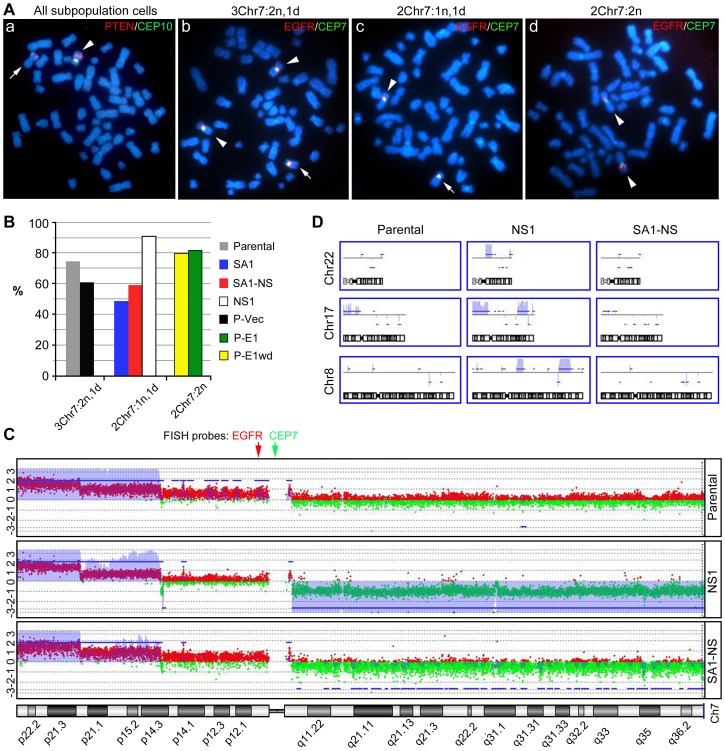
Distinct karyotypes of three subpopulation cells in U251. **A**, representative metaphase FISH pictures of PTEN/CEP10 and EGFR/CEP7 dual probes showing all cells carrying one copy of Chr10, an unknown chromosome with a PTEN translocation, and three cell types differing in their composition of normal and derivative Chr7 (dChr7). *Arrow* points to dChr7; *arrowhead* to normal Chr7. **B**, percentage of majority cells in the parental culture, derived or converted SA or NS subcultures, and the parental culture after lentiviral transductions by pTRIPZ-Vec (P-Vec), pTRIPZ-EFEMP1 with (P-E1) or after withdrawal (P-E1wd) of doxycyclin. **C–D**, comparison of DNA copy number variation in chromosomes 7, 8, 17, and 22 for U251 parental derived or converted NS subcultures of NS1 or SA1-NS, respectively. The Y axis is the log ratio of intensity (the ratio of test sample and normal blood) from comparative genome hybridization. Amplifications or deletions are shown by blue lines above or below the red or green areas, respectively, based on Z-score, and those with marked changes are highlighted in purple.

There are different variants of U251 (see Table S2). The U251 used in this study was previous reported as U251HF, highly tumorigenic and forming invasive intracranial (i.c.) xenografts that displayed GBM histological hallmarks [33]. It has the DNA microsatellite fingerprinting most similar to U251 in the NCI-60 cell line panel [34], carrying all short tandem repeat (STR) markers but the one in the Y chromosome. Our FISH analysis using CEPX/CEPY dual probes verified loss of the Y chromosome in U251HF. As for U251 (NCI), our U251 contains homozygous mutations of PTEN [E242fs*15 (723 724 insTT)] and TP53 (R273H), a derivative Chr7 with amplification of 7p and deletion of 7q, and monosomy 10. In contrast to heterogeneity of cells with Chr7-CNV (see Figure 2), 97% cells in U251 contains one copy of chromosome 10 and two copies “mutant” PTEN (Figure 3A, panel a).

By analyzing over a hundred metaphase cells of U251 and its derived SA and NS clones, we found that 91% of the cells were near diploid, carrying 1-, 2- and 3-copies of Chr7 and a small percentage cells were near tetraploid, carrying 4-, 5- and 6-copies of Chr7. In the parental culture, the majority cell type had two normal and one abnormal 7q-deleted Chr7 (designated as 3Chr7:2n,1d) and two minority subpopulations of cells carrying either two normal Chr7s (2Chr7:2n) or one normal and one 7q-deleted Chr7 (2Chr7:1n,1d) (Figure 3A). The percentage of 2Chr7:1n,1d cells increased in clonal SA subcultures (Figure 2B), and further increased after 1 month in culture under NS conditions (see SA1-NS in Figure 3B). Percentage of 2Chr7:1n,1d cells was as high as 90–92% in NS clones (see NS1 in Figure 3B), as far as they were maintained in NS conditions. The 2Chr7:2n cells, however, remained a minor subpopulation in parental, SA and NS subcultures.

**Figure 4:**
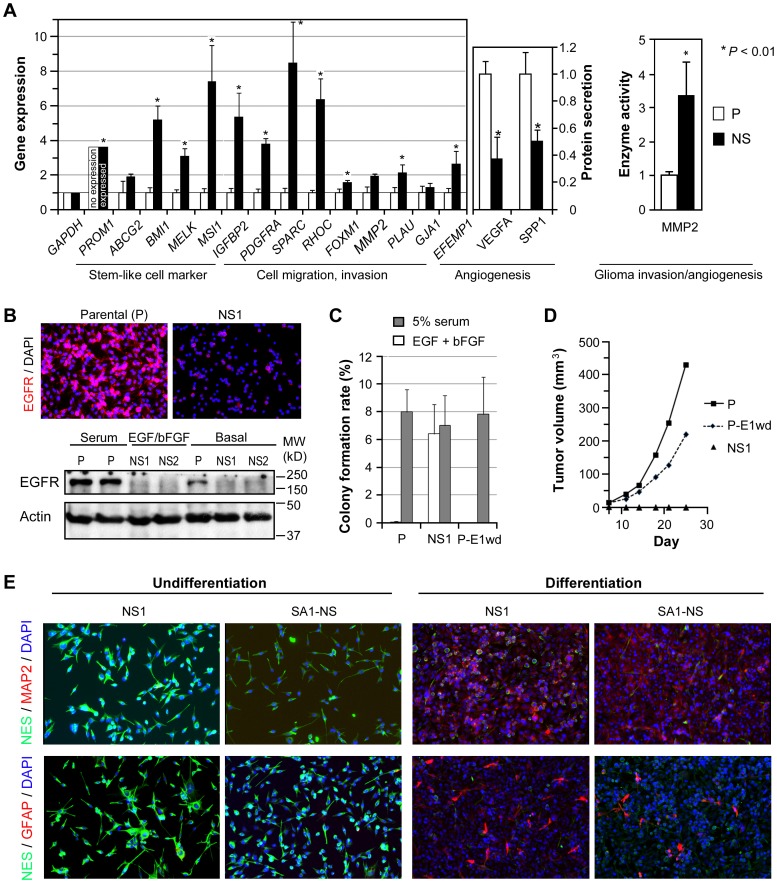
Distinct phenotypes of subpopulation cells in U251. **A**, real-time qRT-PCR (right) quantification of the expression of genes associated with neural stem cell features as well as glioma cell migration and invasion, normalized to *ACTB*, in cells of the same cultures used for zymography, and enzyme immunometric assays (left) for quantification of VEGFA (VEGF-165) and SPP1 (Osteopontin) in conditioned medium, normalized by cell numbers. Bar and line height are mean and SD based on quantification of 3–6 sets of independent cultures. **B**, western blot (top) and immunocytofluorescence (bottom) of EGFR (1∶1000 from Cell Signaling) in U251 parental (P) and two clonal NS lines (NS1 and NS2). **C**, soft agar colony formation assay of U251 parental (P), NS1, and P-E1wd lines. **D**, s.c. tumorigenicity assay of cells described above, with follow-up of tumor growth as described previously [33]. **E**, immunocytofluorescence analysis of NS1 and SA1-NS before and after being subjected to neural stem cell differentiation conditions described in Methods.

In an initially unrelated experiment involving use of lentiviral-mediated expression of EFEMP1, we used U251 parental cells to examine doxycyclin-induced expression of ectopic EFEMP1 (“P-E1” cultures). We found that the 2Chr7:2n cell percentage increased to nearly 80% in P-E1; whereas, cells infected with control vector (see P-Vec in Figure 3B) had a Chr7-cell population equilibrium similar to the U251 parental line, with 3Chr7:2n,1d cells as majority subpopulation (Figure 3B). We have previously reported on the tumor suppressive effect of EFEMP1 in GBM and EFEMP1's function in reducing the EGFR signaling activities in glioma cells [35]. The observed reduction in the proportion of 3Chr7:2n,1d cells in U251from ectopic expression of EFEMP1 is a new finding, consistent with EFEMP1′s suppression of EGFR signaling activity, which is supported by subsequently obtained data showing a high level of EGFR expression in 3Chr7:2n,1d cells (Figure 4B). We then examined the cell composition in P-E1 cultures after withdrawing the doxycyclin-induced EFEMP1 expression (P-E1wd culture). Figure 3B shows that after two passages without EFEMP1 induction (indicated by completely gone of RFP expression), the majority of cells in the P-E1wd culture remained the 2Chr7:2n cells, with a percentage as high as 80%. This observation indicates the robustness of these culture compositions once equilibrium in cell subpopulations has been established.

### Chr7-MS is responsible for inter-conversion of subpopulation cells

We performed comparative genome hybridization (CGH) to determine if there are other DNA-level alterations besides Chr7 that could be specific to certain Chr7-subpopulation cells in U251. If we found such characteristic CNV, it could be used to trace the origins of Chr7 aneuploid cells as coming either from the parental culture or arising *de novo* by Chr7-MS. We compared CGH profiles of U251 parental, NS1, and SA1 re-selected to enrich NS cells by culturing in NS-condiiton for 4 weeks (named as SA1-NS). It is clear that the majority cells in the parental and parentally derived NS subcultures carry one 7q-deleted Chr7 with a nearly complete q-arm deletion and amplification of the distal p-arm (Figure 3C). Figure 3C further demonstrates that the majority cells of the parental culture also carried two copies of normal Chr7, while both NS subcultures derived from parental and SA1 carried one normal Chr7 (the other being a 7q-deleted Chr7), which is consistent with the results of FISH (Figure 2D and Figure 3A).

Comparison of CNV in other chromosomes showed regional amplifications in chromosomes 8, 17, and 22 that were found specifically in NS1 (majority = 2Chr7:1n,1d cells, Figure 3D, middle panel), but were absent in U251 parental (majority = 2Chr7:2n,1d cells) and SA1-NS (majority = 2Chr7:1n,1d cells) (Figure 3D, left and right panels, respectively). In contrast, the CGH profiles of U251 parental and SA1-NS were highly similar for all chromosomes but Chr7. Clearly, the 2Chr7:1n,1d cells in NS1 are descendents of 2Chr7:1n,1d cells pre-existing as the original minority cell subpopulation in U251, while the 2Chr7:1n,1d cells in SA1-NS are descendents of a 3-Chr7:2n,1d cell in U251. The common difference between parental and SA1-NS is the loss of one normal copy of Chr7. These results clearly show that Chr7-MS was responsible for converting a 3Chr7:2n,1d cell into a 2Chr7:1n,1d cell, thereby restoring cell heterogeneity in the glioma culture. The reverse case, where Chr7-MS converts a 2Chr7:1n,1d cell into 3Chr7:2n,1d cell has also been demonstrated by FISH and the functional assays described below.

### Cell subpopulations in U251 have distinct phenotypes

Through our research on U251 and its NS subcultures and EFEMP1-infectants, we obtained three syngeneic cultures, each having a different cell subpopulation type in majority, these being 3Chr7:2n,1d, 2Chr7:1n,1d and 2Chr7:2n cells in parental (P), mixed or clonal NS subcultures (NS, NS1, NS2), and EFEMP1-infectants withdraw the induction of transgene expression (P-E1wd), respectively (Figure 3B). These syngeneic cultures provided us with a unique resource for conducting a study on the phenotypic diversity of subpopulation cells and the benefit a tumor derives from having such cell composition diversity. Important here are the mechanisms that control the dynamics of tumor cell population equilibrium, which could aid our understanding of cancer plasticity and failures in GBM treatment.

First we carried out a comparison of the expression of genes/proteins reported to mark neural stem cells, and to cause changes in cancer cell invasive, proliferative and angiogenic behaviors. We limited molecular analysis to parental (majority = 3Chr7:2n,1d cells) and NS subcultures (majority = 2Chr7:1n,1d cells). Compared to 3Chr7:2n,1d cells, the 2Chr7:1n,1d cells expressed genes of neural stem cells (*PROM1*, *BMI1*, *MELK*, *MSI1*). Immunocytofluorescence of NS1 cells with primary antibodies for CD133, MELK, and BMI1 showed positive staining in the majority cells for MELK and BMI1, but very few cells stained by CD133 (Figure S1), which is consistent with a low expression of CD133 in NS subcultures compared to no expression in parental cells. The 2Chr7:1n,1d cells exhibited a more invasive phenotype by expressing higher levels of the pro-invasive genes (*IGFBP2*, *PDGFRA*, *RHOC*, *FOXM1*, and *PLAU*) and matrix metalloproteinase 2 (MMP2) (Figure 4A), all of which are well-reported for cancers, including GBM [36]–[38]. In contrast, the 3Chr7:2n,1d cells demonstrated a proliferative phenotype with secretion of pro-angiogenic proteins (VEGFA and SPP1) and expression of EGFR (Figure 4A–B) at significantly high levels compared to that by 2Chr7:1n,1d cells.

We then examined the *in vitro* growth of the three syngeneic cultures. Because it is the change of culture medium and attachment that allowed the establishment of NS subcultures with enrichment of cells originally in a low percentage in the parental culture, we used a soft agar colony formation assay to determine each subpopulation's anchorage-independent growth in serum- and/or EGF/bFGF-containing media. All three syngeneic cultures formed colonies at a similar rate in soft agar with medium containing serum (Figure 4C). However, only NS1 (majority = 2Chr7:1n,1d) formed colonies in serum-free NS culture medium, while the P (majority = 3Chr7:2n,1d) and P-E1wd (majority = 2Chr7:2n) failed. This indicated a similar serum-dependent, anchorage independent growth phenotype for 3Chr7:2n,1d and 2Chr7:2n cells, while 2Chr7:1n,1d cells were more flexible with regard to growth conditions.

We carried out subcutaneous (s.c.) implantation of glioma cells (1×10^6^) with 10 independent implantations to examine tumor onset and growth, which are dependent on tumor-cell-induced angiogenesis [35]. Both P (majority = 3Chr7:2n,1d) and P-E1wd (majority = 2Chr7:2n) are tumorigenic in the s.c. xenograft model, with the former showing faster tumor growth than the latter (Figure 4D). In contrast, NS1 (majority = 2Chr7:1n,1d) failed to form s.c. xenografts, even with the follow-up time extended to 44 days. A repeat of the implantation with cells prepared independently at a different time also failed to give a xenograft tumor.

**Figure 5:**
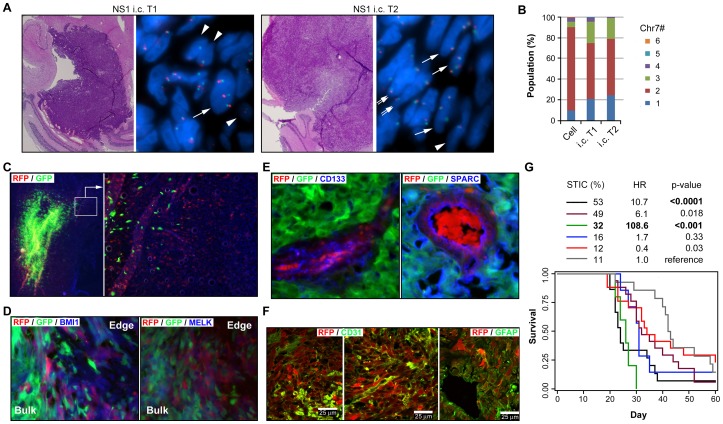
Distinct “go” and “grow” features of two cell subpopulations in U251 with optimal equilibrium benefiting overall tumor growth. **A**, H&E images (2X) and FISH images (100X) of i.c. xenografts derived from NS1. *Arrowhead*, *double* and *single arrow* point to cells with 1, 2, and 3 copies of Chr7, respectively. **B**, comparison of cell population equilibrium in NS1 (*in vitro* culture) and the derived i.c. tumors. **C**, fluorescence images of i.c. xenografts derived from co-implantation of RFP-labeled STIC-enriched U251-NS and GFP-labeled U251 in a 9∶1 ratio. **D–E**, immunofluorescence images of i.c. xenografts from co-implantation of the two lines in a 1∶99 ratio, with purple color marking BMI1 and MELK expression by RFP cells at the tumor boundary and their expression of CD133 and SPARC in vascular mimicry within the bulk tumor mass. **F**, confocal immunofluorescence images of i.c. xenografts derived from 100% RFP cells, with yellow color marking co-localization of RFP with CD31 or GFAP. **G**, Kaplan-Meier survival curves of mice after implanting a mixture of U251 parental and STIC-enriched NS subculture. Adjusted Hazard ratios (HRs) from the stratified analysis and p-value are from Cox regression analyses examining the effect of STIC percentage on survival.

Overall, the results from assays on *in vitro* molecular profiles and growth, and *in vivo* s.c. tumorigenicity, showed distinct growth, angiogenesis, and invasion features that were consistent for each of the subpopulation cell types in U251, with 3Chr7:2n,1d and 2Chr7:2n cells sharing similarity in their serum-dependent growth and ability to form s.c. xenografts, while the 2Chr7:1n,1d cells expressed more invasive proteins and was unable to form s.c. xenografts. The above described neural stem marker expression, sphere-forming phenotype, and the following described re-establishment of tumor hierarchy by a U251-NS clonal line are consistent with features expected from tumor cells with stem-like properties, identified from primary glioma by NS culture [31] or CD133-antibody mediated cell sorting techniques [39]. The high i.c. tumorigenecity for 2Chr7:1n,1d cells further defines its feature as tumor initiating cell (TIC). Other stem-like properties of 2Chr7:1n,1d cells include the stem-like cell multipotency shown in neural stem cell differentiation assay *in vitro* and endothelial differentiation *in vivo*. The former feature was demonstrated by both NS subcultures of parental-line origin or newly generated by Chr7-MS. Both showed increase in expression of glial cell marker GFAP and neuronal cell marker MAP2, and decrease in expression of neural stem cell marker NES, following 1-2 weeks of culturing in differentiation conditions. This expression phenotype was in marked contrast to that of the undifferentiated cells analyzed prior to subjecting them to the differentiation conditions (Figure 4E). The ability of U251-NS cell in forming blood vessel was also shown in below described i.c. xenograft (Figure 5). Hence 2Chr7:1n,1d cell are the stem-like TIC (STIC), the cell subpopulations within primary cultures of malignant glioma with a long-term self-renewal capacity [40].

We also evaluated colony formation rate for both subpopulation cells in U251 by single cell plating of parental cells in adherent culture, limiting dilution assays for NS subcultures in NS and soft agar conditions. The colony formation rate for U251 was 52+/−8% from plating an average of 0.5, 0.7 and 1 cell per well. The sphere formation rate for U251-NS1 was 74%+/−3% from limiting dilution (100, 20, 5 and 1 cell). The colony formation rate in soft agar for U251-NS1 was 6–7% (see Figure 4C). Because NS1 originated from a single soft agar colony, the ability to re-form colonies verified an inherited self-renewing ability, which is frequently referred to as stemness for cancer stem cells. Here our characterization of U251 showed diverse phenotypes of subpopulation cells, which are able to self-renew and inter-convert via Chr7-MS.

### Tumor growth benefits from having cell subpopulations with diverse phenotypes

We further examined the phenotypic characteristics of the STIC from U251 to determine their ability to form tumors in the intracranial (i.c.) xenograft model, to restore subpopulation cell heterogeneity, and to form blood vessels by endothelial trans-differentiation, as previously described for STIC [41], [42]. NS1, which lacked s.c. tumorigenicity from implantation with 1×10^6^ cells (see Figure 4D), formed i.c. xenografts from implantation with 1×10^4^ or 1×10^5^ cells (Figure 5A). FISH analysis of the resulting i.c. xenografts showed a marked increase in the percentage of cells carrying 1 and 3 copies of Chr7 (Figure 5B). The cells with 1 and 3-copies of Chr7 could be found physically near each other, suggesting Chr7-MS of STIC during i.c. xenograft formation.

To demonstrate the *in vivo* infiltrative feature of STIC from U251 shown by *in vitro* assays, we infected U251 parental and NS cells under semi-confluent conditions with two lentiviral vectors, pGIPZ and pTRIPZ, that express green (GFP) and red (RFP) fluorescent proteins, respectively. After 1–2 weeks of culturing, with puromycin elimination of non-infected cells, the infected cells were pooled together for i.c. co-implantation, alone or mixed at various ratios. As shown in Figure 5C, the i.c. xenografts derived from co-implantation with 10% GFP and 90% RFP cells showed a majority of cells expressing GFP and these were located in the center of the tumor mass, whereas cells expressing RFP were found at the peritumoral boundaries. Reversing the co-implantation percentages resulted in an even more striking separation of these two subpopulations, with most of the RFP cells found in the infiltrating tumor boundary. Immunofluorescence analysis verified that the infiltrating RFP cells expressed BMI1 and MELK (Figure 5D), the same as shown in their *in vitro* NS cultures (Figure 4A). These genes have been reported to express in STICs [32].

In i.c. xenografts from co-implantation with 1% RFP and 99% GFP cells, some RFP-expressing cells were associated with blood vessels forming within the main tumor mass_ENREF_10. The presence of naturally autofluorescent erythrocytes[43] in these vascular channels confirmed that they were functionally active, vascular channels formed by tumor cells. Such vessels are commonly found, and are considered to be a pathologic vascularization mechanism in GBM [44], [45]. The RFP cells expressed CD133, a marker frequently used to identify brain-tumor stem-like cells [39], as well as the glioma-associated, secreted protein SPARC [46] (Figure 5E), and the endothelial cell marker CD31, as revealed by confocal microscopy (Figure 5F left). These observations demonstrated endothelial trans-differentiation of STIC from U251, which maintained their glial cell characteristics as shown by expression of GFAP (Figure 5F right).

**Figure 6:**
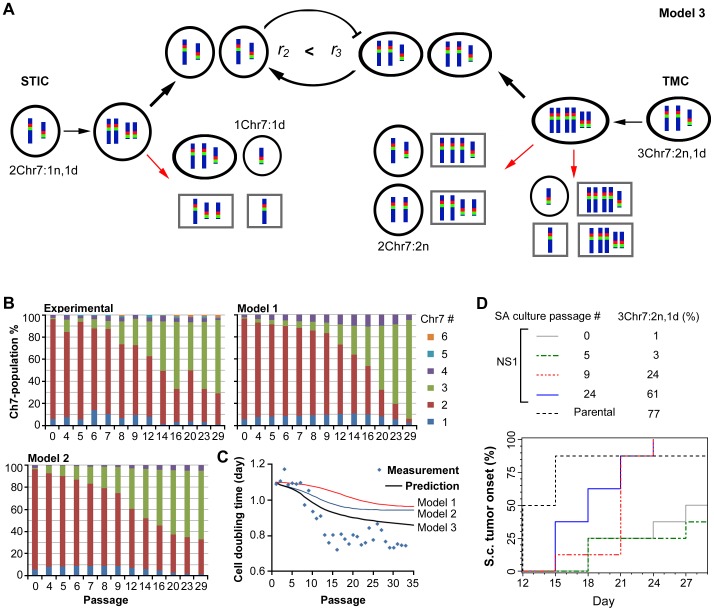
Mathematical modeling of experimental data with changes in population equilibrium from homogeneity to heterogeneity. **A**, schematic illustration of the working mechanism with Chr7-MS resulting in heterogeneous subpopulations and cellular phenotype inter-conversion, with STIC inhibiting growth of TMC and/or TMC stimulating growth of STIC. The Chr7 composition was shown for representative subpopulation cells in U251, based on metaphase FISH.A higher growth rate for TMC (*r*_3_) was shown compared to that for STIC (*r*_2_). Cells marked by black circles were seen in metaphase FISH analysis, suggesting their ability to grow *in vitro*. Cells marked by gray boxes were not seen in metaphase FISH analysis, suggesting they are unable, or have very low ability to grow *in vitro*. **B**, changes in population equilibrium from homogeneity to heterogeneity in NS1 after serial, three-day passages in SA-culture conditions, using the same cell plating density (5×10^5^/100 mm dish), with cell types determined by FISH (experimental) and then modeled using different parameters as detailed in Methods. **C**. changes in cell growth speed as measured or predicted by various mathematical models. **D**, s.c. tumorigenicity assay showing the TMC features of 3Chr7:2n,1d cells converted from STIC by increasing percentage tumor onset (a tumor size of ∼50 mm^3^). The plot with percent uses percents computed via the Kaplan-Meier method. Log-rank test for trend with 3Chr7:2n,1d cells shows two-sided *P*<0.0001.

The above described i.c. xenograft experiments showed the tumor mass-forming cell (TMC) feature in majority cells of U251, which was in striking contrast to less proliferative more invasive phenotype shown in STIC from U251. Such functional diversity between two glioma cell subpopulations with Chr7-CNV would be synergistic to overall tumor growth. Indeed, by analyzing animal survival time in relation to the proportions of U251 subpopulaiton cells with STIC and TMC features, it was found that a ratio of approximately 1 STIC: 2 TMC had the most deleterious effect on mouse survival (Figure 5G). Interestingly, this is also the ratio that showed the highest overall growth speed *in vitro* under SA conditions (Figure 6B), from studying the dynamics of cell composition from homogeneity to heterogeneity, as detailed in the following section.

The overall results described above demonstrated phenotypic differences between TMC and STIC subpopulations of U251. CGH data showed one copy Chr7 and regional amplification in chromosomes 8, 17, and 22 as the major DNA level differences between parental (majority TMC) and NS1 (majority STIC). It is not determined by this study if alterations in chromosomes 8, 17, and 22 are responsible for the different tumor formation properties. However, combining the data of the CGH and the neural stem cell differentiation assays for NS1 and SA1-NS, their involvement in STIC features was excluded.

### Mathematical modeling of cell equilibrium from homogeneity to heterogeneity

By changing culture conditions to NS conditions that favor glioma cells carrying STIC features, we were able to maintain a nearly homogenous culture with STIC, which was a very minor subpopulation in the U251 maintained under SA conditions. Following the observation of autonomous Chr7-MS in single-cell-derived subcultures, which restored the cell heterogeneity seen for the parental culture (Figure 2), we examined the process of restoring culture heterogeneity by returning NS1 with nearly homogenous STIC to SA conditions, taking measurements of cell doubling time and using FISH to analyze the proportion of cells carrying different numbers of Chr7, over 29, 3-day, serial passages. This gives a total of approximately 90 cell divisions. This experiment was repeated once, with averages in cell doubling time and percentage cells varying in Chr7-CNV applied in mathematical modeling, to identify various parameters yielding the observed changes in overall cell growth rate and the subpopulation cell equilibrium. Cell proportions were determined from the FISH data collected for more than 250 cells.

For simplicity, we did not distinguish among cells with different normal and abnormal Chr7 compositions (Figure 6A). A set of mathematical equations describe the time evolution of subpopulations with 1–5 copies of Chr7. Because 92% of cells in NS1 were 2Chr7:1n,1d cells functionally defined as STICs, we considered cells measured by FISH to carrying two copies of Chr7 to be STICs. We called cells carrying three copies of Chr7 in U251 as TMCs, based on their phenotypic characteristics described above and the fact that metaphase FISH data did not show other assortments of normal and abnormal Chr7 (we assumed such cells were possible from Chr7-MS but were unable to survive or grow). For analysis of cell-cell interactions in culture under SA conditions, we ignored the small number of cells in U251 carrying 1 copy of Chr7, with 1Chr7:1d cells observed in metaphase FISH.

We first considered the simplest model (model 1) with no inter-conversion between subpopulations. This model predicted that the subpopulation with the highest growth rate (i.e., TMCs) eventually would sweep away other subpopulations (Figure 6B), which is inconsistent with the experimental observations. We then expanded the model to allow inter-conversions among subpopulations through Chr7-MS (model 2). This model satisfactorily reproduced the observed heterogeneity. Even though the TMC had the fastest growth rate, with Chr7-MS, they always generated other subpopulations, and the culture eventually approached a steady-state distribution.

We experimentally tested the model for its power in predicting changes in cell doubling time over serial passages. There were qualitative differences between the experimental results and outcomes predicted by either model 1 or model 2 (Figure 6C). Therefore we further considered the possibility of interactions between subpopulations. By considering Chr7-MS and interactions between STIC and TMC, specifically, TMC enhancing STIC growth, or STIC inhibiting TMC growth, or both, (model 3), we successfully reproduced the evolution of the subpopulation proportions as for model 2, but further predicted the biphasic behavior of the doubling time (Figure 6C). The nature of the interactions between the subpopulations is not known and would need further study to identify.

The parameters that best fit the dynamic cell population equilibrium measured by CEP7 FISH were shown in Table 1. It showed a higher growth rate of TMC than STIC, suggesting that SA culture conditions favor TMC growth, but Ch7-MS prevented its homogenizing the culture over time. Our model only assumed Chr7-MS, since the experimental data provided only cells with Chr7-CNV. The possibility of other chromosome mis-segregaiton is certainly there, just not being measured. The mis-segregation rates of Chr7-MS in our U251 model are very low (∼0.001 and ∼0.01 per cell division, respectively), which are in the range of aneuploidy rates reported in human cancer cells [47] and yeast [48].

**Table 1:** Parameters that best fit of the percentage time course data.

	r_1_	r_20_	Δ_2_	ρ_2c_	r_30_	Δ_3_	ρ_3c_	r_4_	r_5_	p_2_	p_3_	R_min_
Model 1	1.6	1.6	0	N/A	1.8	0	N/A	1.6	0.02	0	0	0.5
Model 2	1.5	1.6	0	N/A	1.9	0	N/A	0.2	0.1	0.002	0.02	0.1
Model 3	1.8	1.7	0.3	0.2	2.1	0.7	0.4	0.05	0.01	0.001	0.01	0.1

The parameter r_20_ is chosen to have an initial doubling time of 1.1 days.

Given that Chr7-MS is a mechanism for generating culture heterogeneity, the model predicted that a cell colony formed from a single TMC carrying two normal and one deleted Chr7 should also generate a small subpopulation of cells carrying just two normal Chr7 copies (i.e. 2Chr7:2n), in addition to give rise STIC (2Chr7:1n,1d) as shown in Figure 6A. This was indeed proven to be the case by metaphase FISH analysis of single-cell derived clones of U251. These cells were shown to be less responsive to EFEMP1-mediated suppression compared to TMC (3Chr7:1n,1d), which allowed their dominating the culture after a sustained period of culture with expression of ectopic EFEMP1 (see P-E1 in Figure 3B). Overall, the data showed the complexity of cancer cell phenotypes that could be enhanced by the existence of chromosomes with structural abnormality, in addition to their mis-segregations.

### Phenotypic similarity between TMC in the parental culture and that derived from STIC

We have shown above inter-conversion of subpopulation cells in U251 by Chr7-MS, with the STIC phenotype restored by loss of one normal copy of Chr7 from TMC. We also saw the converse, where TMC appeared in culture derived from a single STIC in NS1, whereas the percentage TMC increased after culturing in SA conditions (Figure 6B). To determine if the gain of one copy of Chr7 in STIC was enough to restore the TMC phenotype, we examined the s.c. tumorigenicity of NS1 after it was subjected to SA culture conditions, where the percentage of new STIC increased, based on the discovery of STIC lacking s.c. tumorigenecity (Figure 4D).

We s.c. implanted NS1 after first culturing them under SA conditions for differing numbers of passages (0, 5, 9, 24). FISH showed that the percentages of TMC increased with increase of passage numbers, from 1%, to 3%, 24%, and 61%, respectively. The s.c. xenograft volume measured after implantation (1×10^6^ cells, total 10 implantations) correlated positively with the percentage of TMC prior to implantation (*P*<0.0001) (Figure 6D). Three weeks after implantation, the cell population subjected to over 9 passages under SA conditions, which contained more that 24% TMC, behaved the same as the parental culture in regards to tumor onset (once a tumor reached approximately 50 mm^3^ in size, usually a fast tumor growth will start). In contrast, cells passed only 5 times under SA conditions, which contained fewer than 5% 3Chr7:2n,1d cells, had poor tumor onset. Clearly, the gain of one normal copy of Chr7 by some cells in the STIC population through Chr7-MS restored the TMC phenotype.

### VEGFA overexpression enabled s.c. tumorigenecity of STIC

**Figure 7:**
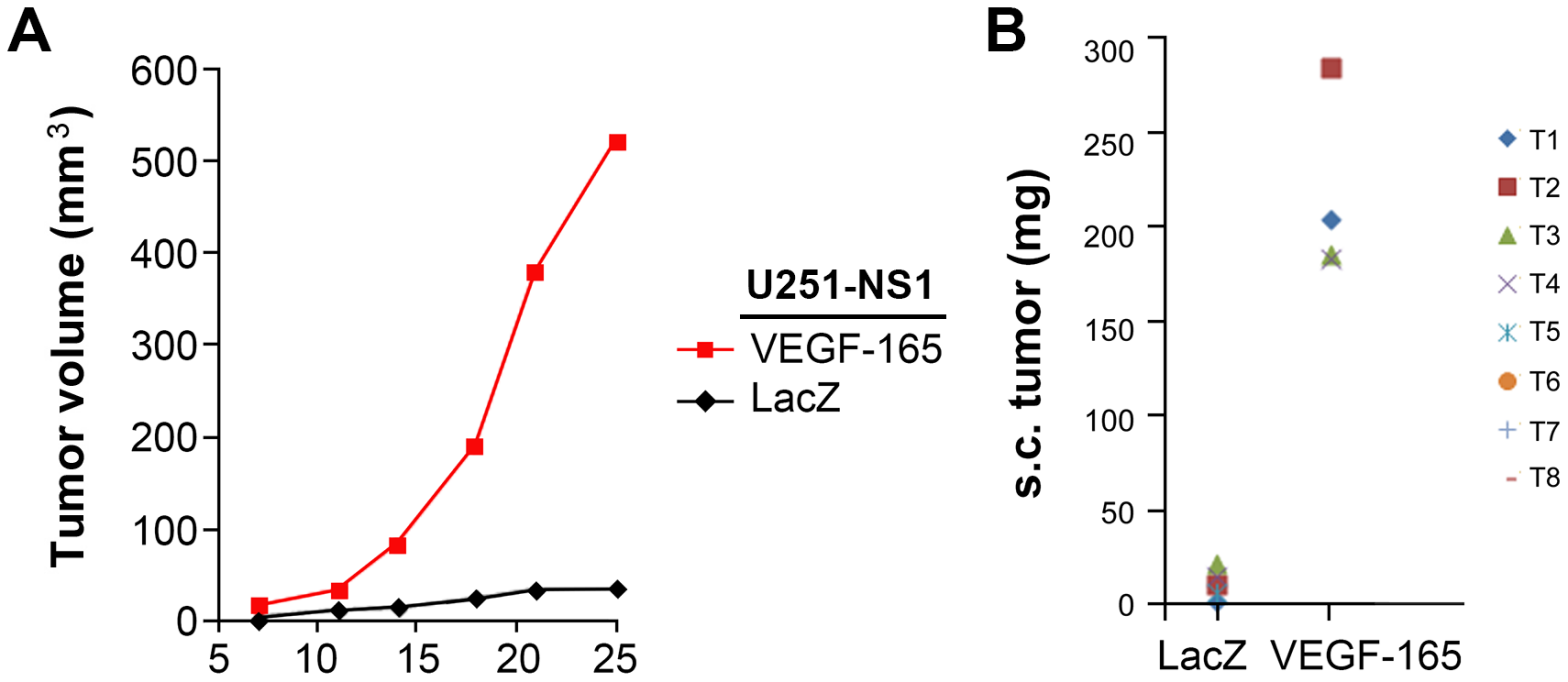
Re-examination of U251-NS1 cell s.c. tumorigenicity after overexpression of VEGFA. U251-NS1 infected with retrovirual vectors of VEGF-165 and LacZ described previously [35] were s.c. implanted in nude mice as described in Materials and Methods.

Failure of NS1 to form tumors in the s.c. xenograft model at a high cell dose (1×10^6^ cells) is in striking contrast to the high i.c. tumorigenecity from lower number of cell (tested on 1×10^3^, 1×10^4^, 1×10^5^ cells). This demonstrates the requirement of orthotopic environment for STIC to grow in the first place. The result of rescuing STIC in s.c. tumorigenecity by a large number of TMC suggests quantitative improvement of tumor microenvironment by TMC. From the observations that overexpression of VEGFA restores s.c. tumorigenecity of U251 that was suppressed by EFEMP1 [35] and that TMC secretes a high level of VEGFA (Figure 4A), we hypothesized that VEGFA overexpression may enable s.c. tumorigenecity of STIC. This hypothesis was supported by results shown in Figure 7, with a high s.c. tumoirgenecity in VEGF-165 transfected NS1.

### Irradiation enhances chromosome mis-segregation of glioma cells

**Figure 8:**
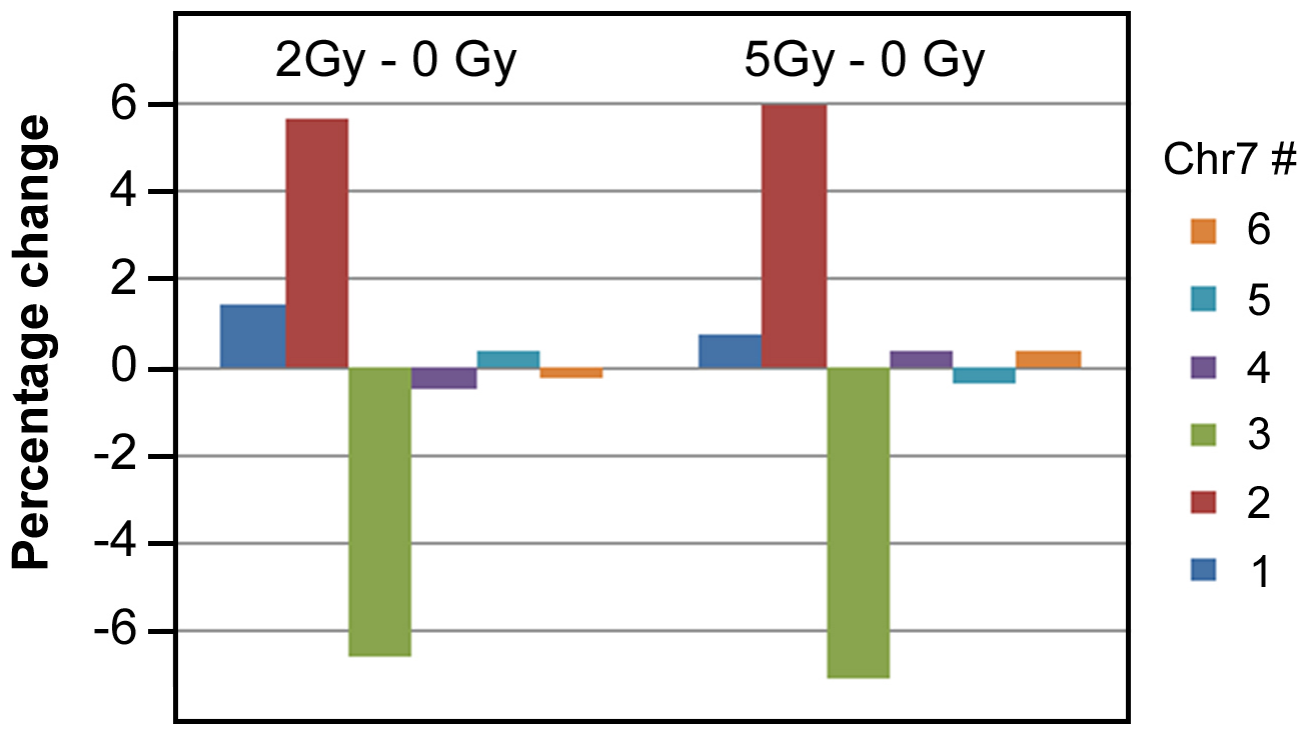
Effect of radiation on Chr7-MS. FISH was performed 1 day after exposure to γ-rays, in single dose, at the 2 and 5 Gy levels.

Radiation is a frontline therapy for gliomas, although it is known to cause various stress responses in the treated cells. We used the defined U251 model and FISH technology to determine if radiation increased the Chr7-MS rate. To provide proof-of-concept data, we performed a simple experiment by exposing semi-confluent U251 cells to single, acute-dose applications at 2 and 5 Gy, and fixing treated cells 24 hours later, for subsequent FISH analysis. The radiation treatments caused an increase in the percentage of cells carrying one or two copies of Chr7, at the expense of cells with three copies of Chr7 (Figure 8). Because the U251 cell doubling time is about 22 hours, the acute radiation effect on changes in the proportions of cell subpopulations suggests an increase in the Chr7-MS rate in the dividing, majority, TMCs. Consistently, metaphase chromosome spreads showed increases of both 2Chr7:2n and 2Chr7:1n,1d cells after irradiation. Taken together with information about the radioresistance of glioma stem-like cells [49] and STIC features of 2Chr7:1n,1d cells described above, our findings describe a new mechanism for explaining glioma radio-resistance and tumor recurrence that is worthy of further investigation.

## Discussion

Non-random distributions of chromosomal gains and losses are common in clinical tumors at both early and late stages, and these are maintained in metastases and cell lines derived from primary tumors. These observations are consistent with an interpretation of chromosome instability as a driver of cancer evolution, progression, and drug resistance through the creation of variable karyotypes, most of them likely inviable, but with selection for the quasi-stable cancer karyotypes that remain [50], [51]. The catalytic role of chromosome instability in cancer development has also been suggested by a theoretical study of cancer progression [52]. It remains an open question in cancer biology whether chromosome instability is involved in maintenance of tumor heterogeneity, defined by cell subpopulations having specific chromosome gains and losses. Our study showed a common existence of Chr7-aneuploid cell subpopulations within gliomas of various malignancies, glioma primary cultures, and cell lines. Finding the re-appearance of specific Chr7-defined cell subpopulations in all single-cell-derived subcultures, of all examined glioma cell lines, provided strong evidence for the involvement of Chr7-MS in the maintenance of tumor heterogeneity in gliomas.

Importantly, by molecular and functional characterization of U251 syngeneic cultures dominated by different cell subpopulations, we discovered a phenotypic divergence of glioma cell subpopulations following re-assortment of Chr7. Interestingly, the behaviors of one subpopulation fit all criteria described for STIC, especially for their pleiotropic cell feature in forming blood vessels [31], [39], [41], [42], in addition to their infiltrative behavior and ability to convert into a highly proliferative cell phenotype by Chr7-MS. The consequence of cell conversion from TMC to STIC by gain an additional Chr7 from Chr7-MS, obviously causes drastic changes in transcription. Comparing to TMC, we have shown STIC to have higher invasiveness, which was correlated with a higher level of expression of pro-invasive genes (Figure 4A), and a peritumoral localization (Figure 5C). The phenotypic transition of cells into a more invasive phenotype has been described as epithelial-mesenchymal transition (EMT), which can be caused by increasing the expression of EMT-responsive genes in both normal and cancer cells. Here, in U251, we show that Chr7-MS causes a similar phenotypic change.

However, the finding of STIC-promoted angiogenesis by Bao et al [53] from studying matched CD133^+^ and CD133^−^ tumor cell populations cultured from D456MG xenografts is not supported by our results from studying functionally defined STIC and TMC from U251. Our finding of VEGFA in promoting tumorigenesis of STIC via angiogenesis is consistent with findings of Oka et al. [54] by studying a line of multipotent, self-renewing cells derived from fresh human GBM. We further showed with evidence from both experimental study and statistical analysis that the angiogenesis could come from proangiogenic factors made by TMCs.

Differential expression of EGFR has been shown in GBM subpopulation cells that were tumorigenic, and increases in EGFR level was shown to be responsible for the highly tumorigenic property [55]. In TMC of U251, overexpression of anti-EGFR protein EFEMP1 almost eliminated the TMC subpopulation during *in vitro* culture of U251-E1 (see Figure 3B). However, activation of the EGFR-mediated growth signal appears not be used by STIC in U251. Activation of developmental signaling pathways of Notch and hedgehog has been shown for STIC [56], [57]. Our finding of a higher NOTCH1 expression in STIC compared to TMC of U251 (data not shown) needs to be further explored to determine if STIC from U251 uses Notch signaling to maintain its growth.

Overall functional characterizations of STIC and TMC subpopulations in U251 provide convincing evidence that the tumor benefits from carrying heterogeneous cell populations. It also suggests that in glioblastoma, both the stem-like cells and mass-forming cells may have the capacity to regenerate the other population through Ch7-MS. This is a much more insidious and complex capability than that originally assumed of a one-way conversion of tumor stem-like cells to tumor mass cells. In having an invasive, pleiotropic cancer cell type, and a less invasive cancer cell type that functions primarily to form the tumor mass, together with cell type inter-conversion by mis-segregation of a specific chromosome, the data we present here support the Darwinian cancer evolution theory.

The finding of interchangeability among cancer cell subpopulations by chromosome instability greatly helps to explain cancer plasticity and robustness. Based on the Darwinian cancer evolution theory, cancer benefits from the co-existence of diverse cell subpopulations within the tumor. The presence of clonal diversity has been shown to predict cancer progression in esophageal adenocarcinoma [3]. Further, the synergistic effect of cell subpopulation diversity on overall tumor growth is evident from the co-implantation experiments using glioma cells expressing the *EGFR* deletion mutant and wild-type EGFR [58]. Both our *in vitro* and *in vivo* experimental data demonstrated the existence of an optimal equilibrium of tumor heterogeneity to overall growth, which was surprisingly similar in the serum adherent *in vitro* culture and the brain tumor orthotopic environment, between the invasive stem-like cells and the proliferative nonstem-like cells. In addition to the experimental data we presented, our mathematical modeling of the experimental data supports the involvement of chromosome instability in opposing clonal homogenization. Furthermore, it suggests that interactions between the cell subpopulations generated a benefit to overall cell growth, which was not directly evident in the experimental data.

Overall, what we have presented here is that Chr7-MS in glioma maintains tumor heterogeneity, which favors overall tumor growth. The possibility of mis-segregation for chromosomes other than Chr7 certainly exists; however, this was not examined during our study. It would be interesting to determine whether there are any additional chromosome mis-segregations involving different chromosomes that also correlate with our observed cell phenotype differences. We believe that our present work opens several new questions to pursue, the answers to which would greatly advance our understanding of cancer evolution, progression and resistance to therapy.

## Supporting Information

Figure S1 Characterization of stem cell marker expression in U251-NS1 cells by immunocytofluorescence in undifferentiated conditions described in Materials and Methods.
(TIF)

Table S1 9-STR DNA Profile of human glioma cell lines used in this study.
(DOC)

Table S2 Comparison of U251 variants by 9 short tandem repeat (STR) markers.
(DOC)
