# Supplementary figures and images for: Tumor-Specific Chromosome Mis-Segregation Controls Cancer Plasticity by Maintaining Tumor Heterogeneity
Source: PLoS One. 2013 Nov 25;8(11):e80898. doi: 10.1371/journal.pone.0080898 (PMC3839911; doi:10.1371/journal.pone.0080898)

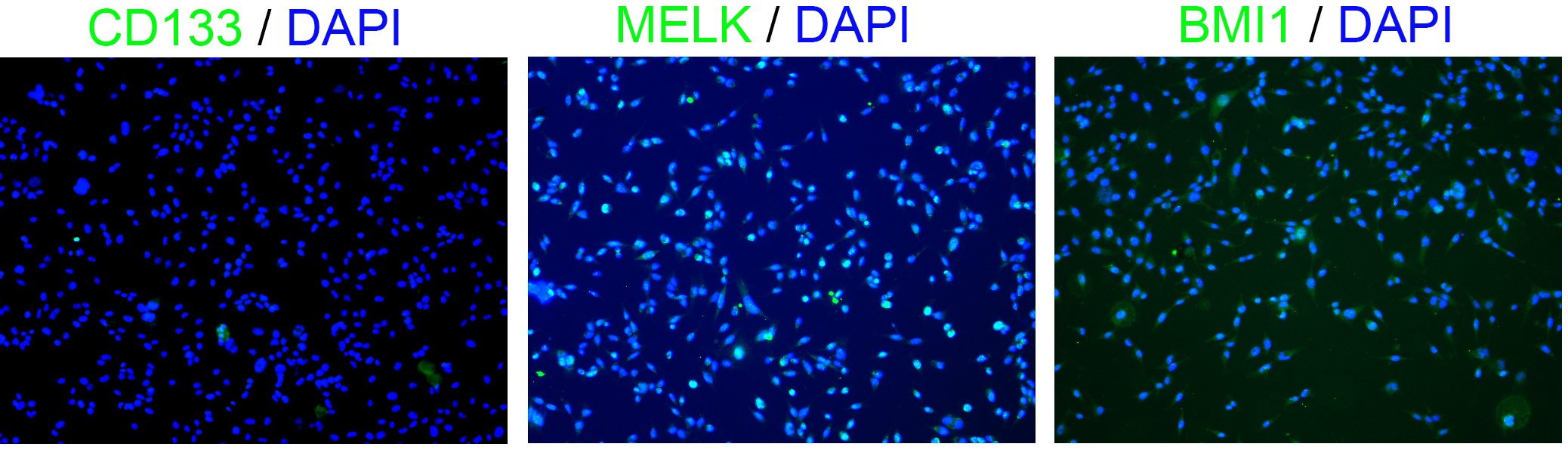

Supplement: Figure S1 — Characterization of stem cell marker expression in U251-NS1 cells by immunocytofluorescence in undifferentiated conditions described in Materials and Methods. (TIF) [file pone.0080898.s001.tif]
