# Supplementary material for: Tumor-Specific Chromosome Mis-Segregation Controls Cancer Plasticity by Maintaining Tumor Heterogeneity
Source: PLoS One. 2013 Nov 25;8(11):e80898. doi: 10.1371/journal.pone.0080898 (PMC3839911; doi:10.1371/journal.pone.0080898)
Supplement: Table S1 — 9-STR DNA Profile of human glioma cell lines used in this study. (DOC) [file pone.0080898.s002.doc]

**Table S1. 9-STR DNA Profile of human glioma cell lines used in this study**

| **Cell line** | A172 | | U87 | | LG11 | LN229 | T98G |
| --- | --- | --- | --- | --- | --- | --- | --- |
| Source | This study | ATCC | This study | ATCC | This study | =ATCC | **=**ATCC |
| Amelogenin | X, Y | X, Y | X, Y* | X | X, Y | X | X, Y |
| CSF1PO | 9, 12 | 9, 12 | 10, 11 | 10, 11 | 12 | 12 | 10, 12 |
| D13S317 | 11 | 11 | 8, 11 | 8, 11 | 11, 12 | 10, 11 | 13 |
| D16S539 | 12 | 12 | 12 | 12 | 8, 14 | 12 | 13 |
| D5S818 | 11, 12 | 11, 12 | 11, 12 | 11, 12 | 11 | 11, 12 | 10, 12 |
| D7S820 | 11 | 11 | 8, 9 | 8, 9 | 11 | 8, 11 | 9, 10 |
| TH01 | 6, 9.3 | 6, 9.3 | 9.3 | 9.3 | 6, 9.3 | 9.3 | 7, 9.3 |
| TPOX | 8, 11 | 8, 11 | 8 | 8 | 8 | 8 | 8 |
| vWA | 16, 20 | 20 | 15, 17 | 15, 17 | 14, 16 | 16, 19 | 17, 20 |

***** U87 is known to be derived from a male donor so the presence of this allele is consistent with the cell line. ATCC, American Type Culture Collection.
