# Supplementary material for: Tumor-Specific Chromosome Mis-Segregation Controls Cancer Plasticity by Maintaining Tumor Heterogeneity
Source: PLoS One. 2013 Nov 25;8(11):e80898. doi: 10.1371/journal.pone.0080898 (PMC3839911; doi:10.1371/journal.pone.0080898)
Supplement: Table S2 — Comparison of U251 variants by 9 short tandem repeat (STR) markers. (DOC) [file pone.0080898.s003.doc]

| **Table S2. Comparison of U251 variants by 9 short tandem repeat (STR) markers** | | | | | | | | | |
| --- | --- | --- | --- | --- | --- | --- | --- | --- | --- |
|  |  |  |  |  |  |  |  |  |  |
| Source | This study | NCI | NCI | JCRB | JCRB | DSMZ | CLS | ECACC | ECACC |
| Cell line | U251HF | U251 | SNB-19^a^ | U-251 MG (KO) | U-251 MG | SNB-19^a^ | U-251 MG | U-251 MG | U-373 MG^a^ |
| Amelogenin | X | X, Y | X, Y | X | X | X,Y | X,Y | X,Y | X |
| CSF1PO | 12, 13 | 12, 13 | 12** | 11, 12 ** | 11, 12 ** | 11, 12 ** | 11, 12 ** | 11, 12 ** | 11,12 |
| D13S317 | 10, 11 | 10, 11 | 10, 11 | 10, 11 | 10,11 | 10,11 | 10,11 | 10,11 | 8 |
| D16S539 | 12 | 12 | 12 | 12 | 12 | 12 | 12 | 12 | 12,13 |
| D5S818 | 11 | 11 | 11,12 | 11 | 11,12 | 11,12 | 11,12 | 11,12 | 12 |
| D7S820 | 10, 12 | 10, 12 | 10* | 10, 12 | 10,12 | 10,12 | 10,12 | 10,12 | 8.2, 12 |
| TH01 | 9.3 | 9.3 | 9.3 | 9.3 | 9.3 | 9.3 | 9.3 | 9.3 | 7, 9.3 |
| TPOX | 8 | 8 | 8 | 8 | 8 | 8 | 8 | 8 | 8 |
| vWA | 16, 18 | 16, 18 | 16, 18 | 16, 18 | 16,18 | 16,18 | 16,18 | 16,18 | 17,18 |
|  |  |  |  |  |  |  |  |  |  |
| ** No allele 13 present in profile. | | |  |  |  |  |  |  |  |
| * No allele 12 present in profile. | | |  |  |  |  |  |  |  |

a- many repositories found out deposited/distributed SNB-19 and U373 MG cell lines were actually U-251 MG cells

b- U118MG and U-138 MG have very similar profiles and most likely originate from the same individual
